# CLIMATE CHANGE: Will Warmer Soil Be as Fertile?

**DOI:** 10.1289/ehp.117-a59

**Published:** 2009-02

**Authors:** Lance Frazer

Growing concern about global climate change has focused increasing research attention on the carbon-regulating role played by soil. Collectively, the Earth’s soils contain more than twice the amount of carbon found in the atmosphere. Scientists at the University of Toronto Scarborough (UTS) now report that global warming may significantly alter soil composition at the molecular level and that such changes could have a major impact on atmospheric levels of carbon dioxide (CO_2_).

Organic matter, the decaying remains of plants and animals, enables soil to support plant life, providing plants and soil microbes with the energy and raw materials they need for growth. Soil microbes facilitate the decomposition of organic matter from litterfall (the leaves, twigs, and other plant materials that fall to the ground), and CO_2_ is a natural by-product of this process. Rising atmospheric temperatures and/or CO_2_ levels are likely to increase photosynthesis and plant productivity, according to the results of several studies over the past two decades; consequently, litterfall is expected to increase. Until now, however, the effects of warming on soil’s molecular composition have been poorly studied. It is therefore unclear to what extent the carbon-containing components of soil matter will accumulate or degrade and thus how much carbon will be sequestered by the soil and how much released into the atmosphere as CO_2_.

In a report published online 23 November 2008 ahead of print in *Nature Geoscience*, environmental chemist Myrna Simpson and her UTS colleagues wrote that rising temperatures would be expected to speed up the decomposition of labile (easily degraded) soil organic compounds such as the carbohydrates from leaf litter, whereas more biochemically resistant carbon-containing structures—such as the lignin from woody tissues and lipids from leaf cuticles—would be expected to remain stable over decades, possibly even centuries. Nevertheless, says Simpson, global warming may change present-day decomposition patterns by altering the soil microbial communities and activities, thus changing the overall flow of carbon into and out of the soil and affecting soil fertility as well. She and her colleagues therefore attempted to discern how such a transition might unfold from the molecular perspective.

Their experiment took place in a moist mixed forest in southern Ontario close to the UTS campus. The site was separated into a control plot and a treatment plot, each measuring 4 meters by 4 meters. The soil temperature was unaltered in the control plot, while the test plot was heated through a series of steel pipes running through the ground. Temperature differences between the control and treatment plots were 3.5–4.5ºC in the summer, spring, and autumn, and 5–6ºC in the winter. The team carried out a detailed analysis of molecular changes in soil organic matter using a combination of gas chromatography/mass spectrometry, nuclear magnetic resonance, and total organic carbon analysis.

They found that the activity of some microorganisms increased in the warmer soil, resulting in a faster degradation of carbohydrates and other labile components. However, the study also showed that soil fungi numbers and activity also increased in the warmer soil, with a corresponding rise in abundance of lignin-derived compounds (reflecting decomposition by the fungi). “The implication of the increased degradation of lignins is that less carbon remains in the soil solid phase, and more CO_2_ is being released from the soil into the atmosphere,” Simpson says.

Conversely, certain other recalcitrant molecular structures—such as the alkyl structures found in the waxy coating or cuticles of leaves—remained resistant to decomposition and accumulated in the warmer soil. Thus, says Simpson, as global temperatures warm and more organic matter from litterfall is added to the soil, less of that matter will be in a form that’s easily used by microbial decomposers in the soil, including many soil bacteria that are needed to sustain robust plant life. “In other words, the soil’s molecular carbon composition is shifting [toward] a form that is not usable to microbes or plants, and more of the remaining carbon is being derived from leaf cuticles that persist in the soil,” she says.

“[The Simpson study] raises some interesting points,” says Jerry Hatfield, who directs the U.S. Department of Agriculture National Soil Tilth Laboratory. “Research already indicates that as you move from northern climes to southern and temperatures increase, it becomes more difficult to increase the organic material in soil.”

Howard Epstein, an associate environmental science professor at the University of Virginia, urges caution in interpreting the study’s findings. “The experiments in this paper provide very short-term results,” he says, “and often initial responses are not sustainable in the future. Systems adjust, and it is unreasonable to imply that litterfall from plants will increase with warming as suggested and remain high, [and] it is also unreasonable to assume that decomposer communities will not adapt to changing dead organic substrates.”

Indeed, says Simpson, increased litter-fall itself may be a short-term observation, “because as nutrients in the soil decline, so will plant growth.” The take-home message, she says, is that global warming may change the composition of soil organic matter, leading to shifts in the ability of soil to sustain plant and microbial life.

The authors note that their observations in the moist, sandy loam soil of the Canadian forest may not apply to soil bio-geochemical processes in dry areas or soils with high clay contents. Moreover, they did not measure nutrient levels or CO_2_ releases directly, and Simpson notes there is no set soil temperature increase that is expected to occur uniformly around the world. Nevertheless, she says, “we found an increased degradation of some soil organic matter components whose end-point is CO_2_, so CO_2_ levels would increase with higher rates of degradation.”

Until now, soil-climate research has focused heavily on the total amount of carbon in the soil, but Simpson believes the more relevant issue may be changes in the carbon-based molecular structures contained in the soil’s organic matter and how such changes will ultimately affect both microbial and plant life. “To understand the soil–climate interactions better, we will need more soil research to focus on the molecular level with an eye toward predicting both short- and long-range changes in the system,” she says.

## Figures and Tables

**Figure:**
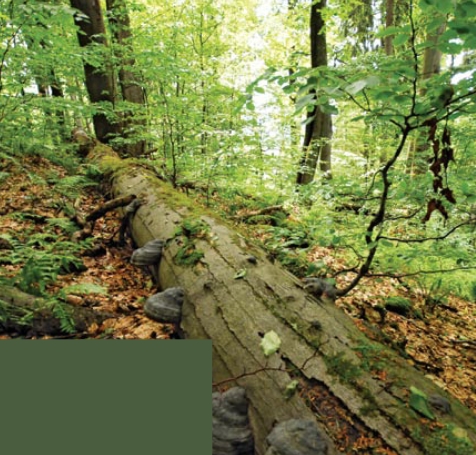
Decomposition of organic matter may change with rising soil temperatures.

